# Mathematical model insights into arsenic detoxification

**DOI:** 10.1186/1742-4682-8-31

**Published:** 2011-08-26

**Authors:** Sean D Lawley, Molly Cinderella, Megan N Hall, Mary V Gamble, H Frederik Nijhout, Michael C Reed

**Affiliations:** 1 Department of Mathematics, Duke University, 130 Science Drive, Durham, NC 27708, USA; 2 Department of Biology, Duke University, 125 Science Drive, Durham, NC 27708, USA; 3 Department of Epidemiology, Mailman School of Public Health, Columbia University, 722 West 168th Street, New York, NY 10032, USA; 4 Department of Environmental Health Sciences, Mailman School of Public Health, Columbia University, 722 West 168th Street, New York, NY 10032, USA

## Abstract

**Background:**

Arsenic in drinking water, a major health hazard to millions of people in South and East Asia and in other parts of the world, is ingested primarily as trivalent inorganic arsenic (iAs), which then undergoes hepatic methylation to methylarsonic acid (MMAs) and a second methylation to dimethylarsinic acid (DMAs). Although MMAs and DMAs are also known to be toxic, DMAs is more easily excreted in the urine and therefore methylation has generally been considered a detoxification pathway. A collaborative modeling project between epidemiologists, biologists, and mathematicians has the purpose of explaining existing data on methylation in human studies in Bangladesh and also testing, by mathematical modeling, effects of nutritional supplements that could increase As methylation.

**Methods:**

We develop a whole body mathematical model of arsenic metabolism including arsenic absorption, storage, methylation, and excretion. The parameters for arsenic methylation in the liver were taken from the biochemical literature. The transport parameters between compartments are largely unknown, so we adjust them so that the model accurately predicts the urine excretion rates of time for the iAs, MMAs, and DMAs in single dose experiments on human subjects.

**Results:**

We test the model by showing that, with no changes in parameters, it predicts accurately the time courses of urinary excretion in mutiple dose experiments conducted on human subjects. Our main purpose is to use the model to study and interpret the data on the effects of folate supplementation on arsenic methylation and excretion in clinical trials in Bangladesh. Folate supplementation of folate-deficient individuals resulted in a 14% decrease in arsenicals in the blood. This is confirmed by the model and the model predicts that arsenicals in the liver will decrease by 19% and arsenicals in other body stores by 26% in these same individuals. In addition, the model predicts that arsenic methyltransferase has been upregulated by a factor of two in this population. Finally, we also show that a modification of the model gives excellent fits to the data on arsenic metabolism in human cultured hepatocytes.

**Conclusions:**

The analysis of the Bangladesh data using the model suggests that folate supplementation may be more effective at reducing whole body arsenic than previously expected. There is almost no data on the upregulation of arsenic methyltransferase in populations chronically exposed to arsenic. Our model predicts upregulation by a factor of two in the Bangladesh population studied. This prediction should be verified since it could have important public health consequences both for treatment strategies and for setting appropriate limits on arsenic in drinking water. Our model has compartments for the binding of arsenicals to proteins inside of cells and we show that these comparments are necessary to obtain good fits to data. Protein-binding of arsenicals should be explored in future biochemical studies.

## I. Introduction

Arsenic in drinking water is a major health hazard to millions of people in South and East Asia and in other parts of the world [1,2]. Long term arsenic exposure has been linked to cancer, heart disease, neuropathies and neurological sequelae, and to deficits in intelligence in children [3,4]. Arsenic in water is normally ingested primarily as trivalent inorganic arsenic (iAs), which then undergoes hepatic methylation to methylarsonic acid (MMAs) and a second methylation to dimethylarsinic acid (DMAs). Each step involves a reduction from pentavalent to trivalent form. While the intermediate trivalent form of MMA is known to be highly toxic [5-7], the pentavalent form, DMA^V^, is more readily excreted in urine and facilitates elimination of As. This is evident in AS3MT deficient mice which demonstrate substantially higher As retention in tissues [8].

The purpose of our collaborative project between epidemiologists and mathematical modelers is to investigate, through modeling, various proposed nutritional supplements that could increase the speed of arsenic methylation in hepatic cells. S-adenosylmethionine (SAM), a metabolite of methionine, is the universal methyl group donor. The SAM concentration is influenced by the folate cycle and the rest of one-carbon metabolism via the methionine synthase reaction that remethylates homocysteine to methionine. It is known both from experimentation [9] and from modeling [10] that an increase in folate status increases the concentration of SAM in hepatic cells. Thus one might predict that increasing folate status would increase the rate of methylation of iAs and this has been verified for folate-deficient individuals in Bangladesh [11,12]. Other proposed supplements are the products of other methylation pathways that might cause those pathways to be down regulated leaving more methyl groups available for methylating arsenic. Since the biochemical pathways are complex, highly regulated, and interconnected, it is not easy to guess what the results of such supplementation will be.

**Figure 1:**
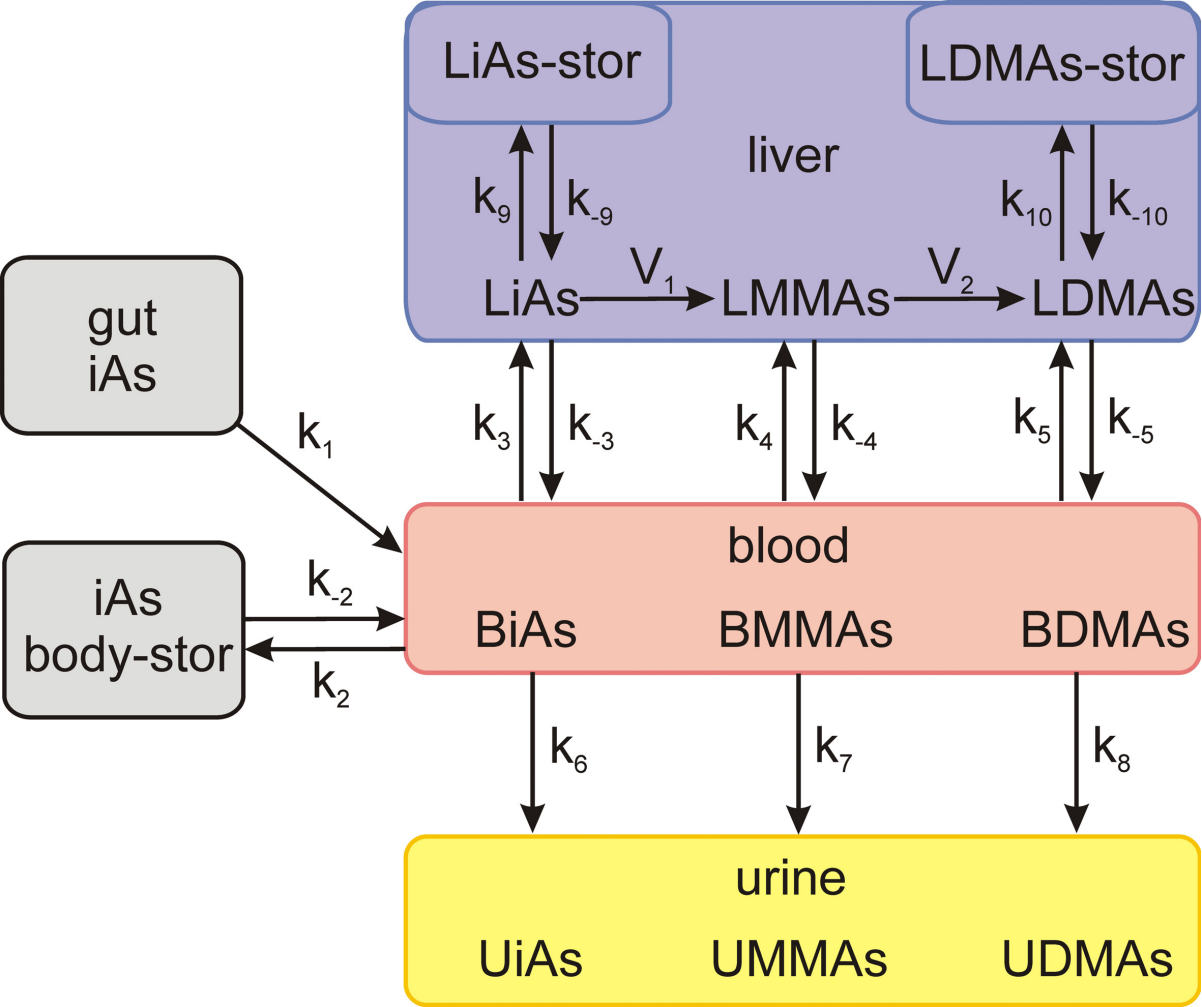
**A schematic description of the model**. Rate constants, abbreviations, differential equations, and difficult modeling issues are discussed in Methods.

Because iAs and its methylated metabolites, MMAs and DMAs, are not measured in the livers of human subjects but in blood and in urine, it is important to have a whole body model that connects arsenic metabolism in the liver to the blood and urine concentrations of iAs, MMAs, and DMAs. This paper presents such a model (Figure 1). We chose the parameters for the methylation reactions from the biochemical literature and adjusted the transport parameters so that the model accurately predicts the single dose experiments in [13]. Necessarily the model contains various simplifications of the complex biochemical processes by which various arsenicals are transported between compartments and methylated and stored in human livers. We describe some of these simplifications in the Discussion.

We use the model to explore three different data sets. First we show that the model, with no changes of parameters gives excellent fits to the time courses of urine excretion of the arsenic metabolites in the multiple dose experiments in [14]. Second, we study the extensive data of Gamble et al. [11,12] who showed that folate supplementation of folate-deficient individuals in Bangladesh increases DMA in urine and decreases total blood arsenic by 14%. The model confirms the 14% decrease in the blood. In addition the model predicts two other consequences (not measured in Bangladesh) of folate supplementation: (1) that liver arsenicals decrease by 19% and (2) other body stores decrease by 26%. In addition, the model predicts that arsenic methyltranferase is, on average, upregulated by a factor of two in these individuals. These predictions, which are the main results of our modeling, should be tested experimentally. Third, we show that, with some changes of parameters, the model gives good fits to experiments on human cultured hepatocytes.

There are a number of other pharmacokinetic models of whole body arsenic storage and methylation. Mann et al. [15] created a whole body model with many tissue compartments for hamsters and rabbits and then extended the model for use with human data [16]. Further work by this group created a whole body model for mice [17]. The model by Yu et al. [18] was used to make predictions but was not compared to experimental data. Kenyon et al. [19], and Easterling et al. [20], created detailed models for methylation and transport into and out of hepatocytes so that they could be used to understand the cell culture experiments in [21] on rat and human hepatocytes. And, finally, El-Marsi et al. [22] extended this model to the whole body case. Each of these models treats methylation somewhat differently than we do and differently from each other.

## II. Methods

A schematic diagram of the model is given in Figure 1. All of the indicated transport velocities and reactions are assumed to have linear dependence on their substrates except for the two methylation reactions. The full names of the variables in the model are indicated in Table 1 and the differential equations satisfied by the variables are indicated below. Table 2 contains the values of all rate constants. Following the differential equations we discuss the main modeling issues.

**Table 1:** Variables in the Model (*μ*M)

gut	inorganic arsenic in the gut
BiAs	blood inorganic arsenic
BMMAs	blood monomethyl arsenic
BDMAs	blood dimethyl arsenic
bodystor	storage of inorganic arsenic in non-liver tissues
LiAs	liver inorganic arsenic
LiAs-stor	liver storage of inorganic arsenic
LMMAs	liver monomethyl arsenic
LDMAs	liver dimethyl arsenic
LDMAs-stor	liver storage of dimethyl arsenic
UiAs	urinary inorganic arsenic
UMMAs	urinary monomethyl arsenic
UDMAs	urinary dimethyl arsenic

**Table 2:** Constants*.

gut = 1	blood = 3	liver = 2	store = 30	volumes (liters)
$r_{gb}=\frac{1}{3}$	*r_sb _*= 10	$r_{lb}=\frac{2}{3}$		volume ratios
*k*_1 _= .11	*k*_2 _= .9	*k*_-2 _= .01		gut to blood, blood to body store (hr^-1^)
*k*_3 _= 7	*k*_-3 _= 1	*k*_9 _= .1	*k*_-9 _= .01	iAs, blood to liver, liver to liver store (hr^-1^)
*k*_4 _= .1	*k*_-4 _= .1			MMAs, blood to liver (hr^-1^)
*k*_5 _= .2	*k*_-5 _= .1	*k*_10 _= .1	*k*_-10 _= .1	DMAs, blood to liver, liver to liver store (hr^-1^)
*k*_6 _= .253	*k*_7 _= .17	*k*_8 _= .85		iAs, MMAs, DMAs, blood to urine (hr^-1^)
*K_m _*= 4.6	*V_mαx _*= .7	$K_{i}^{\text{iAs}}=40$	$K_{i}^{\text{MMA}}=1.26$	methylation, iAs → MMAs (*μ*M or *μ*M/hr)
*K_m _*= 3	*V_mαx _*= .7	$K_{i}^{\text{iAs}}=40$		methylation, MMAs → DMAs (*μ*M or *μ*M/hr)

* for definition of the rate constants see Figure 1 and the text.

$$\begin{array}{lll} \frac{d\left[\text{gut}\right]}{dt} & =input\left(t\right)-k_{1}\left[\text{gut}\right]\; & \text{(1)}\\ \frac{d\left[\text{BiAs}\right]}{dt} & =k_{1}r_{gb}\left[\text{gut}\right]+k_{-2}r_{sb}\left[\text{bodystore}\right]+k_{-3}r_{lb}\left[\text{LiAs}\right]\;-\left(k_{3}+k_{2}+k_{6}\right)\left[\text{BiAs}\right]\; & \text{(2)}\\ \frac{d\left[\text{BMMAs}\right]}{dt} & =k_{-4}r_{lb}\left[\text{LMMAs}\right]-\left(k_{-4}+k_{7}\right)\left[\text{BMMAs}\right]\; & \text{(3)}\\ \frac{d\left[\text{BDMAs}\right]}{dt} & =k_{-5}r_{lb}\left[\text{LDMAs}\right]-\left(k_{5}+k_{8}\right)\left[\text{BDMAs}\right]\; & \text{(4)}\\ \frac{d\left[\text{bodystore}\right]}{dt} & =k_{2}r_{sb}^{-1}\left[\text{BiAs}\right]-k_{-2}\left[\text{bodystore}\right]\; & \text{(5)}\\ \frac{d\left[\text{LiAs}\right]}{dt} & =k_{3}r_{lb}^{-1}\left[\text{BiAs}\right]-k_{-3}\left[\text{LiAs}\right]+k_{-9}\left[\text{LiAs}-\text{store}\right]-k_{9}\left[\text{LiAs}\right]-V_{1}\left(\left[\text{LiAs}\right],\;\left[\text{LMMAs}\right]\right)\; & \text{(6)}\\ \frac{d\left[\text{LiAs}-\text{store}\right]}{dt} & =k_{9}\left[\text{LiAs}\right]-k_{-9}\left[\text{LiAs - store}\right]\; & \text{(7)}\\ \frac{d\left[\text{LMMAs}\right]}{dt} & =k_{4}r_{lb}^{-1}\left[\text{BMMAs}\right]-k_{-4}\left[\text{LMMAs}\right]+V_{1}\left(\left[\text{LiAs}\right],\;\left[\text{LMMAs}\right]\right)-V_{2}\left(\left[\text{LiAs}\right],\;\left[\text{LMMAs}\right]\right)\; & \text{(8)}\\ \frac{d\left[\text{LDMAs}\right]}{dt} & =k_{5}r_{lb}^{-1}\left[\text{BDMAs}\right]-k_{-5}\left[\text{LDMAs}\right]+V_{2}\left(\left[\text{LiAs}\right],\;\left[\text{LMMAs}\right]\right)+k_{-10}\left[\text{LDMAs}-\text{store}\right]-k_{10}\left[\text{LDMAs}\right]\; & \text{(9)}\\ \frac{d\left[\text{LDMAs}-\text{store}\right]}{dt} & =k_{10}\left[\text{LDMAs}\right]-k_{-10}\left[\text{LDMAs}-\text{store}\right]\; & \text{(10)}\\ \frac{d\left[\text{UiAs}\right]}{dt} & =3k_{6}\left[\text{BiAs}\right]\; & \text{(11)}\\ \frac{d\left[\text{UMMAs}\right]}{dt} & =3k_{7}\left[\text{BMMAs}\right]\; & \text{(12)}\\ \frac{d\left[\text{UDMAs}\right]}{dt} & =3k_{8}\left[\text{BDMAs}\right]\; & \text{(13)}\\ & \; & \text{(14) } \end{array}$$

### IIA. Methylation reactions

The velocity of the first methylation step, *V*_1_, is given by

(1) $$V_{1}=\frac{V_{max}\left[LiAs\right]}{\left(K_{m}+\left[LiAs\right]\right)\left(1+\frac{\left[LiAs\right]}{K_{si}}\right)\left(1+\frac{\left[LMMAs\right]}{K_{i}}\right)}$$

We take the value of the Michaelis-Menten constant *K_m _*= 4.6 *μ*M for arsenic (+3 oxidation state) methyltransferase (AS3MT) from [23]. This reaction shows substrate inhibition by LiAs as well as inhibition by the product LMMAs. We take the substrate inhibition constant *K_si _*= 1.26 *μ*M from [24]. While product inhibition of the AS3MT enzyme is known to occur, *K_i _*values are not currently available, so we choose *K_i _*= 40 *μ*M for the enzyme described in [25]. We note that different methyltransferases have been identified in different species [26]. For the second methylation step, *V*_2_, we use the formula

(2) $$V_{2}=\frac{V_{max}\left[MMAs\right]}{\left(K_{m}+\left[MMAs\right]\right)\left(1+\frac{\left[LiAs\right]}{K_{i}}\right)}.$$

We chose the *K_m _*= 4.6 *μ*M as for the first step. This reaction from LMMAs to LDMAs is also inhibited by LiAs and we choose the inhibition constant to be *K_i _*= 40 *μ*M from [25]. The value of *V_mαx _*= 1 *μ*M/hr for both reactions was chosen by fitting the Buchet data (see below).

### IIB. Storage Compartments

Since we are not focusing on the distribution of arsenic compounds in different body tissues, our model has only a single whole-body storage compartment that represents all the non-liver tissues. Arsenic compounds are measured in units of *μ*M in the model so the volumes of compartments are important because the ratios of volumes appear in the differential equations when arsenic compounds are transported from one compartment to another. The volumes assumed and their ratios are give in Table 2.

We include in our model a liver storage compartment for iAs and a liver storage compartment for DMAs because we found that including such compartments was necessary to obtain excellent fits to data. There are many reasons to believe that arsenic compounds bind to proteins in the liver; in fact, such binding is one of the likely modes of arsenic toxicity [27,28]. These bound arsenicals are not available for methylation. The models in [19] and [20] also used liver storage compartments for arsenic compounds in their pharmacokinetic models for the *in vitro *experiments in [21]. We found that including a compartment for MMAs liver storage was not necessary to obtain excellent fits to the data. Thus, in order to keep our model as simple as possible, we did not include such a compartment, even though there surely is some binding of MMAs to proteins.

### IIC. Arsenic input

We assume that single oral doses of arsenic become available in the gut over a six minute period and are then transported with linear kinetics (*k*_1_) into the blood. The value of *k*_1 _= .11/hr listed in Table 2 is the transport rate of arsenic trioxide, which is the usual compound that is dosed. In the MMAs and DMAs dosing experiments described below, *k*_1 _is changed to 2/hr and .125/hr respectively. There is evidence that the different arsenic metabolites are transported at different rates from the gut to the blood [29], and these different values appeared naturally when we fit the data in Buchet et al. (1981a). We also use a background dose of 7 *μ*g per day as measured by Buchet et al. [13]. For the discussion of chronic exposure and the Gamble data in the Results Section, we take the input to the gut to be constant so that the total daily input is 300 *μ*g.

### IID. Arsenic output

Notice that the differential equations for the urinary metabolites, UiAs, UMMAs, UDMAs, have an extra factor of 3, which is the assumed volume of the blood plasma in liters. Thus, unlike the other variables in the model, the urinary variables are in micromoles rather than micromolar so that we can easily compare to data. To obtain the model data points at the end of each time interval, we accumulate each of the arsenic species in the urine since the previous data point and then divide by the time between data points to get a rate of excretion to compare to the measured rates in urine.

### IIE. Tuning of parameters

In the experiments of Buchet et al. [13], a 500 *μ*g dose of iAs was given and the time courses of iAs, MMAs, and DMAs in the urine were measured. In a separate experiment, a 500 *μ*g dose of MMA was given and the time courses of MMAs and DMAs in the urine was followed. Finally, a 500 *μ*g dose of DMAs was given and the time course of DMAs in the urine were followed. We exploited this wealth of data as follows. First we considered just the DMAs experiment, since only the parameters *k*_5_, *k*_-5_, *k*_10_, *k*_-10_, *k*_8 _and rate from gut to blood are involved. After tuning these parameters to get the experimental DMAs output curve, we then considered the MMAs experiment. Here five new parameters were involved, *k*_4_, *k*_-4_, *k*_7_, the *V_mαx _*of the second methylation step, and the rate of MMAs transfer from gut to blood. After tuning these parameters to obtain the MMAs and DMAs output curves in this case, we then considered the full model with iAs input and compared to the output curves for iAs, MMAs, and DMAs.

**Figure 2:**
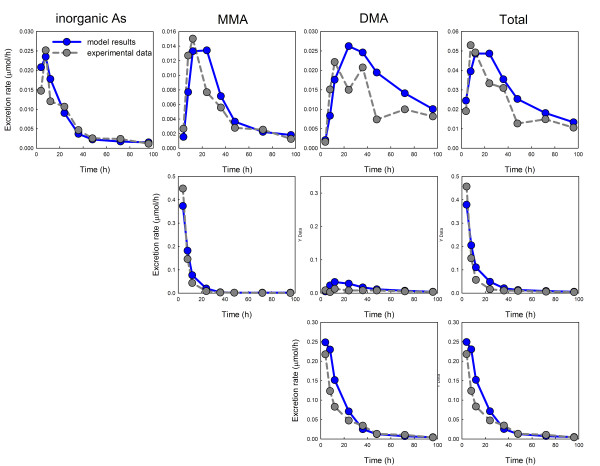
**Fits of model results to the single dose data in Buchet et al. **[13]. Model results are given in blue, experimental data in grey (micromoles/hr in the urine). In the first row volunteers were given 500 *μ*g of inorganic arsenic. In the second row they were given 500 *μ*g of MMAs. In the third row they were given 500 *μ*g of DMAs.

After we tuned the parameters, the model gave excellent fits, both quantitatively and qualitatively, to the iAs experiments, the MMAs experiments, and the DMAs experiments for single doses reported in Buchet et al. [13] over the full 100 hour time course. It is known that individuals show large variation in their ability to methylate arsenic [30,31]. The experimental data shown in Figure 2 consists of averages over only 3 individuals (iAs or MMAs) or four individuals (DMAs). Thus one would not expect perfect fits for any model. Below, we use the model, validated for the Buchet et al. [13] single dose experiments, to explore its fit to other experimental studies.

## III. Results

First we compare model predictions with the repeated dose experiments in Buchet et al. [14]. Next, we use the model to study and interpret the data obtained by Gamble et al. [11,12] who measured blood and urine arsenic levels in the Bangladesh population both with and without folate supplementation. Finally, we show that using the same model, but with different parameters, we can match the *in vitro *experiments in [21].

### IIIA. Data from repeated dose experiments

**Figure 3:**
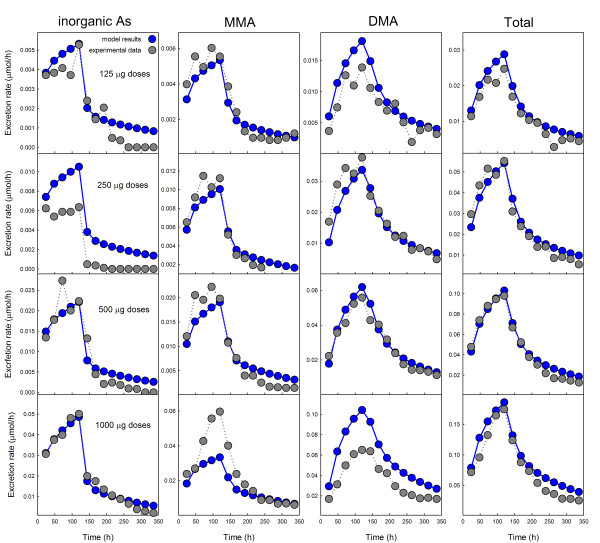
**Comparison of model results to the repeated dose experiments in Buchet et al. **[14]. Model results are given in blue, experimental data in grey (micromoles/hr in the urine). In the first, second, third, and fourth rows, volunteers were given 5 daily doses of 125 *μ*g, 250 *μ*g, 500 *μ*g, 1000 *μ*g, of inorganic arsenic, respectively. The four columns show the time courses of inorganic arsenic, MMAs, DMAs, and total arsenic in the urine, respectively, over 14 days.

In the multiple dose experiments of Buchet et al. [14], four volunteers were given five daily doses of arsenic at the levels 125 *μ*g, 250 *μ*g, 500 *μ*g, and 1000 *μ*g, respectively. Their urine was collected over a 14 day period and the number of *μ*moles of inorganic arsenic, MMAs, and DMAs were measured. These amounts were divided by the number of hours since the last sample to obtain a current rate of metabolite excretion in *μ*mole/hr at each time point. The results are indicated by the grey dots in Figure 3, where the four rows correspond to the doses of 125 *μ*g, 250 *μ*g, 500 *μ*g, and 1000 *μ*g respectively and the columns are the excretion rates for inorganic arsenic, MMAs, DMAs, and total arsenic respectively. In each case the blue dots show the excretion rates produced by our mathematical model using the set of parameters determined by fitting the single dose data in Buchet et al. [13], as described in Methods. No parameters were changed in any of the 12 model experiments shown in Figure 3. Note that the scales on the y axes differ in the different panels of Figure 3.

The model predictions of the time courses of excretion rates are quite close to the experimentally observed time courses, except for two cases. The first methylation step was quite a bit faster for the volunteer who received the 250 *μ*g dose than the model predicts. And, the second methylation step was slower for the volunteer who received the 1000 *μ*g dose than the model predicts. In all four cases, the match to the time course of total arsenic excretion was excellent. It should be pointed out that the experimental results in each row are measurements for a single individual and it is well known that individuals can differ significantly in their arsenic storage and methylation capacities. Thus it is remarkable that our simple model, with the same choice of parameters, fits the data so well for all four individuals who received different repeated doses (Figure 3) as well as those receiving a single dose (Figure 2).

**Figure 4:**
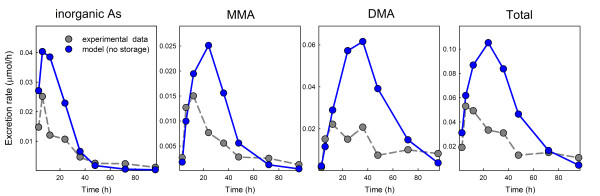
**The storage compartments are necessary**. Model results are given in blue, experimental data in grey (micromoles/hr in the urine). If all storage compartments are removed, the model predictions differ considerably from the data in Buchet et. al., 1981a, for the experiments with a 500 *μ*g dose of iAs. Compare to row 1 in Figure 2 where the model includes all three storage compartments.

An important result of our modeling is that we found it necessary to include storage compartments for iAs and DMAs in the liver. In all cases in Figure 3 the concentration of inorganic arsenic drops rapidly after the fifth and final dose, and then has a long flat tail, as do the concentrations of MMAs and DMAs. This suggests that free arsenic is quite rapidly methylated and cleared and that the long flat tails correspond to slow leakage of arsenic from storage compartments. But is this storage in extrahepatic tissues or in liver cells? It is difficult to tell from the urine excretion rates in Figure 3. However, the *in vitro *data [21] on human liver cells have similar long flat tails suggesting that there is storage in the liver cells. In fact, there is strong experimental evidence in [27] and [28] that arsenicals bind substantially to proteins. Liver storage compartments for arsenic compounds were used by Kenyon [19] and Easterling [20] in modeling the human hepatocyte data [21]. We experimented with all possible combinations of storage compartments, body storage, and liver storage for iAs, MMAs, and DMAs. We found that to get good fits to the data that we used to tune the model (Figure 2), we needed both the body storage of iAs and the storage of iAs and DMAs in the liver cells. If any one of these storage compartments is omitted, the model predictions differ considerably from the data. We found that we did not need a storage compartment for MMAs in order to obtain excellent fits to the data, so we did not include it in the model, even though it is likely there is some binding of MMAs to liver proteins also. Figure 4 shows the model predictions for the third row in Figure 3 (5 repeated doses of 500 *μ*g daily) with the storage compartments removed. Note that the model curves go to zero before 200 hours while the data have long flat tails.

### IIIB. The effect of folate supplementation on arsenic methylation

Gamble et al. [11,12] have shown that folate supplementation of folate-deficient individuals in Bangladesh significantly increased arsenic methylation and decreased total blood arsenic. The *a priori *hypothesis of Gamble was that the mechanism is as follows: 5-methyltetrahydrofolate is a substrate for the methionine synthase reaction through which homocysteine is remethylated to methionine that is then converted to S-adenosylmethionine (SAM). Thus one would expect the SAM concentration to be positively correlated with the folate status of the individual and, indeed, a correlation coefficient of 0.74 between serum folate and liver SAM was reported in [32]. Reed et al. [10] showed, using a mathematical model of folate and methionine metabolism, that SAM concentration (as percent of normal) in liver cells is a linear function of liver total folate (as percent of normal) with a slope of about 0.9. A later more comprehensive mathematical model, described in [33], gives a similar result but the slope is computed to be 1 (unpublished). One would thus expect that folate supplementation would increase SAM in folate-deficient individuals. SAM is the universal methyl donor in liver cells, so as SAM concentration rises more methyl groups should become available for AS3MT. Folate supplementation should increase the percentage of arsenic in the DMAs form and, since DMAs is excreted more rapidly than MMAs or iAs, one would expect the rate of arsenic excretion to rise. The purpose here is to make quantitative calculations about these effects and to compare the model predictions with the results measured by Gamble et al. [11,12] in Bangladesh. Another part of Gamble's hypothesis is that folate supplementation should lower SAH and thus lower the inhibition of AS3MT, but this hypothesis is not tested here.

In the definitions of *V*_1 _and *V*_2 _in the Methods we assumed that the concentration of SAM was constant and the effect of SAM was included in *V_mαx_*. We now make the dependence on SAM explicit:

$$\begin{array}{ll} V_{1} & =\frac{V_{max}[LiAs]}{\left(K_{m}+[LiAs])(1+\frac{\left[LiAs\right]}{K_{si}})(1+\frac{\left[LMMA\right]}{K_{i}}\right)}\left(\frac{\alpha [SAM]}{K_{m}+[SAM]}\right)\\ V_{2} & =\frac{V_{max}[MMA]}{\left(K_{m}+[MMA])(1+\frac{\left[LiAs\right]}{K_{i}}\right)}\left(\frac{\alpha [SAM]}{K_{m}+[SAM]}\right). \end{array}$$

We take the *K_m _*for SAM to be 11.8 *μ*M from [23], and we will choose *α *to be such that the last factor is equal to one when SAM has a "normal" concentration. Thus the methylation reactions are as described in the Methods when SAM is normal (corresponding to the folate supplemented population) but will be slower when SAM is below normal (corresponding to the folate-deficient population before supplementation). We remark that, for simplicity, we are leaving out the inhibition of the methyl transferase reactions by S-adenosylhomocysteine and the homeostatic effects of the long-range inhibitions discussed in Nijhout et al. [34].

Unfortunately, measurements of liver SAM in the folate supplemented and folate-deficient populations are not available, so we must estimate them from other studies. The classical studies of Finklestein [35,36] found a mean liver SAM concentration of 83.6 *μ*M in rats and a range of 60-160 *μ*M on different diets. On the other hand, Selhub [37] found SAM concentrations in human livers to be in the range 20 - 60 *μ*M. In our previous modeling studies, where necessarily most of the parameters are determined from rat data, we have often assumed that liver SAM is 60 *μ*M. If we assume that "normal" SAM is 60 *μ*M, then the constant *α *= 1.1967. If we assume that "normal" SAM is 40 *μ*M, then the constant *α *= 1.2950. Below we argue that liver SAM in the folate-deficient population should be about $\frac{1}{4}$ of normal. The results described below are not very different if we assume normal SAM is 60 *μ*M and folate-deficient SAM is 15 *μ*M or if we assume normal SAM is 40 *μ*M and folate-deficient SAM is 10 *μ*M. So we will choose SAM = 60 *μ*M and *α *= 1.1967.

The next question is how to estimate liver SAM in the folate-deficient population. Gamble et al. [11] studied 194 folate-deficient individuals in Bangladesh; their mean plasma folate was approximately 8 nmol/L. Pfeiffer et al. [38] found a mean folate level in the U.S. population (after folate fortification) of approximately 32 nmol/L in plasma. So, the folate-deficient group studied by Gamble et al. had only 1/4 the observed serum folate concentration found in the U.S. after fortification. Clifford et al. [39] show that serum and liver folate are highly correlated and that when serum folate goes up by a factor of 3, liver folate goes up by a factor of 2.5. In addition, Min et al. [32] show that when liver folate goes up by a factor of 3.5, then liver SAM also goes up by a factor of approximately 3.5. This is consistent with our modeling studies in which we found that SAM concentration (as percent of normal) in liver cells is a linear function of total liver folate (as percent of normal) with a slope of 0.9.-1. Taken together, these studies suggest that, in the physiological range, it is reasonable to assume that liver SAM increases proportionally to serum folate with a slope of about one. Thus since the folate-deficient group in Bangladesh had only $\frac{1}{4}$ normal folate, we estimate that they also had approximately $\frac{1}{4}$ normal SAM.

The Bangladesh population has been continuously exposed to arsenic in drinking water and it would be reasonable to assume that their average expression levels of AS3MT are substantially higher than those of the Belgian volunteers [13] on which the model was tuned in the Methods Section. Unfortunately, there are no data to support this assumption and no way of directly comparing the AS3MT expression levels in the two groups. There are data that suggest a modest increase in AS3MT expression in rats after exposure to arsenic in drinking water [40] and in [41] it is suggested that the high percentage of DMAs in the urine of a population exposed to high levels of arsenic is probably due to upregulation of AS3MT. It is well known that methylation rates vary widely within and between human populations [31,30]. These large differences likely depend on genetic background, nutritional status, and environmental exposure and it is difficult to determine how to untangle these causes without more human genetic studies. Therefore, we experimented with the model to see what increase in AS3MT (corresponding to an increase of *V_mαx_*) in the methylation reactions would give the mean blood levels of the arsenic metabolites and the mean arsenic excretion profiles observed by Gamble et. al. [11,12] in the folate-deficient Bangladesh population. We found that doubling the AS3MT expression level gave an almost perfect fit to the Gamble data before supplementation (Table 3).

**Table 3:** Measured and model percentages of arsenicals for the Bangladesh folate-deficient population before folate supplementation.

	inorganic As	MMAs	DMAs
blood (Gamble)	26%	40%	34%
blood (model)	26%	39%	35%
urine (Gamble)	15%	13%	72%
urine (model)	15%	15%	69%

To summarize, we modified the model described in the Methods as follows. We included explicitly the dependence of the methylation reactions on SAM and chose *α *so that the new factor in equations (1) and (2) equals one if SAM concentration is normal. To obtain the profile of the folate-deficient Bangladesh population, we lowered SAM from 60 *μ*M to 15 *μ*M and we doubled *V_mαx _*from 1 *μ*M/hr to 2 *μ*M/hr. Therefore the methylation reactions are as given in equations (3) and (4) below. All other parameters are identical to those in the Methods.

(3) $$V_{1}=\frac{2V_{max}\left[LiAs\right]}{\left(K_{m}+\left[LiAs\right]\right)\left(1+\frac{\left[LiAs\right]}{K_{si}}\right)\left(1+\frac{\left[LMMAs\right]}{K_{i}}\right)}\left(\frac{15\alpha }{K_{m}+15}\right)$$

(4) $$V_{2}=\frac{2V_{max}\left[MMAs\right]}{\left(K_{m}+\left[MMAs\right]\right)\left(1+\frac{\left[LiAs\right]}{K_{i}}\right)}\left(\frac{15\alpha }{K_{m}+15}\right).$$

Table 3 shows that the model percentages of the arsenicals in the blood and in the urine correspond very closely to those reported by Gamble et al. ([12], their Figure 3) for the folate-deficient population before supplementation.

Gamble et al. [11,12] measured the arsenicals in the blood and urine after 12 weeks of folate supplementation (400 *μ*g/day). In the model, we represent the result of folate supplementation by returning the value of [SAM] from 15 *μ*M to the normal value of 60 *μ*M. Table 4 compares the model results with those observed by Gamble et al. [11,12].

**Table 4:** Measured and model percentages of arsenicals for the Bangladesh folate-deficient population after 12 weeks of folate supplementation.

	inorganic As	MMAs	DMAs	total (% change)
blood (Gamble)	23%	37%	40%	-14%
blood (model)	22%	33%	45%	-13%
urine (Gamble)	10%	11%	79%	
urine (model)	11%	11%	77%	
liver (model)				-19%
body-stor (model)				-26%

Folate supplementation not only shifted the balance of arsenicals towards DMA in the blood and the urine, it also reduced the total arsenic in the blood (by 14% in Bangladesh population and by 13% in the model). After folate supplementation, the percentages of the urinary metabolites are very similar in the model and the Gamble data. It is particularly interesting that total liver arsenic (free plus storage) drops by 19% in the model after folate supplementation and that the body store drops by 26% in the model after folate supplementation. These are quantities that could not be measured in Bangladesh. Thus, the model results suggest that folate supplementation for this folate-deficient population may have even greater health benefits than are suggested by the measured 14% drop in blood arsenic. Other model results (not shown) also mimic the clinical results. For example, urinary DMAs increases substantially after one week of supplementation before descending to a more modest increase from baseline at 12 weeks. Therefore, the model gives strong support to the hypothesis that folate supplementation is helpful for the folate-deficient population because increasing folate increases SAM dramatically for those individuals. Since their SAM concentrations are relatively low (near the *K_m _*of SAM for AS3MT) increasing SAM has a large effect on the rate of methylation. It is not entirely clear that folate supplementation would increase methylation of arsenic for a folate-sufficient population because higher than normal SAM concentrations are well over the *K_m _*of SAM, so the methylation reactions are already saturated. However, folate supplementation might also lower SAH, which would release some of the inhibition of AS3MT and therefore increase methylation.

### IIIC. Arsenic exposure in human hepatocytes

The dynamic behavior of all three arsenicals, iAs, MMAs, and DMAs have been studied in *in vitro *experiments with cultured rat and human hepatocytes [21]. Therefore it was of interest to see whether a modification of our model corresponding to their experimental conditions would give predictions that were close to the experimental curves.

We modified the model by eliminating the urine compartment, input from the gut, and body storage. The word "blood" is now replaced in Figure 1 by the word "medium" and the word "liver" is replaced by the word "cells." The differential equations are the same as those given in the Methods except that many of the rate constants (*k*_1_, *k*_6_, *k*_7_, *k*_8_, *k*_10_, *k*_-10_) are now equal to 0. In addition, *r_cm _*replaces *r_lb_*. There is no body store but we do have storage in the medium (see below) between free and bound iAs (rate constants *k*_2 _and *k*_-2_) and this means that *r_sb _*= 1. The constants are indicated in Table 5.

**Table 5:** Constants for the reduced model containing medium and cells.

medium = 0.5	cells = 0.0005	*r_cm _*= 0.001		volumes (ml), volume ratio
*k*_2 _= 0.4	*k*_-2 _= .07			iAs, medium to medium store (hr^-1^)
*k*_3 _= 0.45	*k*_-3 _= 0.5	*k*_9 _= 0.3	*k*_-9 _= 0.05	iAs, medium to cells, cell to cell store (hr^-1^)
*k*_4 _= 0.2	*k*_-4 _= 0.05			MMAs, medium to cells (hr^-1^)
*k*_5 _= 0.015	*k*_-5 _= 0.2			DMAs, medium to cells (hr^-1^)
*K_m _*= 4.6	*V_mαx _*= 22	$K_{i}^{\text{iAs}}=40$	$K_{i}^{\text{MMA}}=1.26$	methylation, iAs→ MMAs (*μ*M or *μ*M/hr)
*K_m _*= 4.6	*V_mαx _*= 2.75	$K_{i}^{\text{iAs}}=40$		methylation, MMAs → DMAs (*μ*M or *μ*M/hr)

We include storage in the cells for iAs as we did in the full model but we didn't need storage of DMAs in the cells. We include storage in the medium because the collagen coated well plates used by [21] are likely to bind iAs because of the sulphur groups on collagen. We note that the model in [20] uses cell storage for iAs as we do. The Styblo wells had a volume of 0.5 ml and there were approximately (.25) × 10^6 ^cells in each culture. Assuming each cell has volume 2 × 10^-9^, which is reasonable, then the volume of cells is 0.0005 ml and the volume ratio of cells to medium is 0.001.

We note that the rate constants in Table 5 are somewhat different from the rate constants in Table 2. This is not surprising since transport between the medium and the cells will be different than transport between the blood and hepatocytes in whole liver, the medium store and the body store are different, and the cultured cells are likely to be different from hepatocytes in whole liver. However, the *K_m _*and the *K_i _*values are the same.

**Figure 5:**
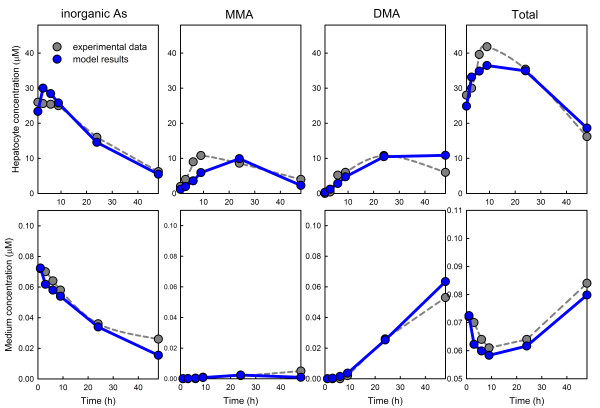
**Comparison of model predictions to data on human hepatocytes**. Experimental data points are in grey, model predictions are in blue. 0.1 *μ*M was introduced into the medium at *t *= 0. The data from Figure 1 of Styblo et al. [21], has been converted into micromolar.

In the experiments in [21], arsenic was introduced into the medium at *t *= 0 and the times courses of iAs, MMAs, DMAs, and total arsenic in the medium and in the cells were measured. The grey dots in Figure 5 are the experimental values for an initial arsenic concentration of 0.1 *μ*M. The blue dots are the model predictions and match the experimental points well. In particular they capture the convex and concave shapes of total arsenic curves in the cells and the medium.

## IV. Discussion

We have developed a relatively simple mathematical model for arsenic storage, methylation, and excretion into the urine (Figure 1). We lumped all non-liver body storage compartments together because our main interest is methylation in the liver and the dynamics of urinary excretion. All transport, excretion, and storage reactions are linear in the model, but the methylation reactions in the liver are Michaelis-Menten (including inhibition) and are based on experimental measurements of *K_m _*and *K_i _*values. The other parameters were systematically tuned by using the data on ingestion of single doses of DMAs, MMAs, and iAs in Buchet et al. [13], and excellent fits to the data were obtained (Figure 2). We remark that many previous whole body models of arsenic metabolism (discussed in the Introduction) try to fit the functions that give the cumulative amounts of iAs, MMAs, and DMAs in the urine, while we fit the rates of excretion. We do this for three reasons. First, the data itself come as amount excreted in a time period, i.e. a rate. Second, it is much easier to fit the cumulative curves since both the data and the model curves will be increasing and saturate. It is much more difficult to fit the rate curves, and thus fitting the rate curves is a more stringent test of the model. Finally, there is information in the rate curves that it hard to see in the cumulative curves; for example, the rate curves show long, flat tails indicative of slow leakage out of storage compartments.

Various aspects of the biochemistry of arsenic metabolism have been greatly simplified in this whole body model. We assume that aresenic is ingested as arsenic trioxide; in fact, relatively small amounts of other forms of arsenic are also ingested. We ignore completely the reduction steps from pentavalent forms to trivalent forms in the liver that use glutathione. And, finally, we ignore many of the complex regulatory mechanisms in one-carbon metabolism that affect methylation, such as the inhibition of the methylation reactions by S-adenosylhomocysteine and the inhibition of glycine methyltranferase by 5-methyltetrahydrofolate. The fact that our model fits three different sets of data so well suggests that these simplifications are reasonable in a whole body model. In future work on competing methylation pathways the full details of the biochemistry will be included.

In Section IIIA, we compared the predictions of the model (with no changes of parameters) to the multiple dose experiments in Buchet et al. [14]. The model predictions correspond very well to the experimental urine excretion curves at all four dose levels (Figure 3). A feature of our model is the use of storage compartments in the liver corresponding to the reversible binding of arsenicals to proteins. The experimental urine excretion rates in Figures 2 and 3 drop rapidly after the dose or final dose but then have long flat tails, particularly for iAs and DMAs. This suggests that free arsenic is quite rapidly methylated and excreted and that the long flat tails are due to the slow leakage of arsenicals from storage compartments. This idea is not new; Kenyon et al. [19] and Easterling et al. [20] used liver storage compartments in their simulations of the *in vitro *data in [21]. We assumed liver storage for iAs and DMAs, but not for MMAs, since we did not need storage of MMAs to get good fits to and predictions of the data. Figure 4 shows what our model predictions of the Buchet et al. single dose data [13] would be if all storage compartments were removed. The fit is poor, to say the least. We experimented with all possible combinations of body storage and liver storage and found that we needed both liver stores and the body store to get good fits and predictions.

In Section IIIB we compared model predictions to the measurements of Gamble et. al. [11,12] of the effects of folate supplementation on the blood and urine levels of arsenicals in a Bangladesh population of folate-deficient individuals. All parameters in the model remained the same except that we had to include explicitly the dependence of the methylation rates on SAM concentration and we had to make assumptions about how folate-deficient the population was. Details and justifications of these assumptions are given in IIIB. The model predictions correspond very closely to the Gamble measurements of all three arsenicals in the blood and in the urine both before and after folate supplementation. The model predicts a drop in total blood arsenic after supplementation of 13% while a drop of 14% was measured by Gamble et al., 2007. In addition, the model predicts a drop in total arsenic in the liver of 19% and a drop in body storage of 26%. These quantities could not be measured, of course, in the population. Thus the model suggests that the health benefits of folate supplementation might be even greater than those suggested by the 14% drop in measured blood arsenicals.

One interesting result of our analysis is that to fit the Gamble data we had to increase the *V_mαx _*of the methylation reactions by a factor of two. This suggests strongly that arsenic methyltransferase is upregulated by a factor of two in the Bangladesh population studied as compared to the Belgian volunteers in the Buchet studies. Methylation rates vary widely within and between human populations [31,30] and these large differences likely depend on genetic background, nutritional status, and environmental exposure. Determining the causal influence of these factors on methylation is important for designing intervention strategies. This will likely require more human genetic studies, more population-based studies relating nutritional status to methylation capacity, and more modeling studies to assist in generating hypotheses and in the interpretation of data.

Supplementation with vitamin B12 will raise the rate of the methionine synthase reaction and therefore should also increase SAM. However, in the randomized controlled trial by Gamble et al. [12], B12-deficient individuals were excluded from the study cohort, so we do not have data on B12 status from the same population. We have shown in a modeling study [10] that B12 status has a much smaller effect on SAM concentration than does folate status. The reason is that in B12-deficient individuals 5-methyltetrahydrofolate will build up (the methyl trap) driving the methionine synthase reaction faster and partially compensating for the lack of B12. Consistent with this explanation is the finding of Selhub [37] that folate deficiency has a much greater effect on homocysteine concentration than does vitamin B12 deficiency.

In Section IIIC we modified the model by eliminating the urine compartments and the body storage so we could see if the resulting model would be sufficient to capture the results of the *in vitro *experiments of Styblo et al. [21] on human hepatocytes. With some changes of transport parameters (the *K_m _*and *K_i _*values for the methylation reactions were kept the same), the model predictions were quite close to the Styblo results (Figure 5).

As described in the Introduction, the goal of our collaborative project is to investigate, through modeling, various proposed nutritional supplements that could increase the speed of arsenic methylation in hepatic cells. This will require us to extend our existing models of one-carbon and glutathione metabolism ([42,10,33]) to include many of the parallel methylation reactions that use SAM as a substrate. The methyltransferase reactions are all inhibited (with different *K_i _*values) by the product, S-adenosylhomocysteine. Other elaborate control mechanisms (see [34]) are known to exist. The elucidation of the control mechanisms (both genetic and metabolic) by which the cell regulates the balance of flux between the various methyltransferases is an important biological problem. By understanding this question through mathematical modeling, we hope to be able to evaluate current proposals and make new proposals for nutritional intervention strategies for populations exposed to chronic doses of arsenic. The whole body model in this paper is the first step in our project, since it will enable us to connect the dynamics of the methylation reactions in the liver to the presence of arsenicals in the blood and the urine where they are usually measured.

## Competing interests

The authors declare that they have no competing interests.

## Authors' contributions

SL and MC wrote the initial code for the model and researched the literature for enzyme kinetic data and made the figures. MG and MH, initiated the project, provided data from their Bangladesh field studies, and gave advice on the epidemiology and biochemistry of arsenic. HN and MR further developed the model and jointly wrote the manuscript with advice from MG and MH. All authors have read and approved the final manuscript.
